# Use of Shockwave intravascular lithotripsy for the treatment of symptomatic and severely calcified superior mesenteric artery stenosis

**DOI:** 10.1186/s42155-021-00243-5

**Published:** 2021-06-14

**Authors:** Oscar Balboa Arregui, Carmen Seoane Pose, María Balboa Alonso, Teresa Bolaño Pampín

**Affiliations:** 1 Division of Vascular and Interventional Radiology, Department of Radiology, Hospital Universitario de León, León, Spain; 2 Division of Vascular and Interventional Radiology, Department of Radiology, Hospital Clínico Universitario de Santiago de Compostela, A Coruña, Spain; 3 Department of Emergencies, Hospital San Juan de Alicante, Alicante, Spain

**Keywords:** Intravascular lithotripsy, Superior mesenteric artery stenosis, Chronic mesenteric ischemia

## Abstract

**Background:**

We present the use of intravascular lithotripsy as a treatment for highly calcified superior mesenteric artery stenosis.

**Case presentation:**

A 67-year-old diabetic man had chronic postprandial abdominal pain and weight loss. Computed tomography angiography revealed highly calcified stenosis of the superior mesenteric artery. Selective angiography confirmed severe stenosis. A Shockwave lithotripsy balloon catheter was successfully used via brachial access to modify calcified plaque and increase vascular lumen. After 12 months of follow-up the patient had gained weight and had no abdominal postprandial pain.

**Conclusion:**

Intravascular lithotripsy could be considered a new treatment modality to modify calcified lesions in the visceral arteries. More controlled studies are needed to demonstrate the efficacy, safety and feasibility of this new technology.

**Level of evidence:**

4, Case Report

## Background

Clinical manifestations of chronic mesenteric ischemia (CMI) may present widespread symptoms such as postprandial abdominal pain and weight loss when there is a 60% to 75% reduction in blood flow. Superior mesenteric artery (SMA) stenosis is a common finding in elderly patients with atherosclerosis. Hansen found that the prevalence of significant stenosis in subjects over 65 was 17.5% (Hansen et al. 2004), with abdominal angina depending on the collaterality of SMA. Image tests as computed tomography angiography (CTA) or lateral projection angiography are needed to visualize the narrowing of the SMA. If CMI is diagnosed there are two therapeutic options: surgical revascularization or percutaneous transluminal angioplasty (PTA) with or without stenting (Sreenarasimhaiah 2007). PTA, less invasive procedure, has associated complications such as thrombosis, restenosis and dislocation of stent (Sakorafas et al. 2008).

Intravascular lithotripsy (IVL) therapy was applied as a definitive treatment for vascular stenosis in a patient who had postprandial abdominal pain and weight loss due to intense calcification and severe stenosis in the proximal segment of SMA.

## Case presentation

**Fig. 1:**
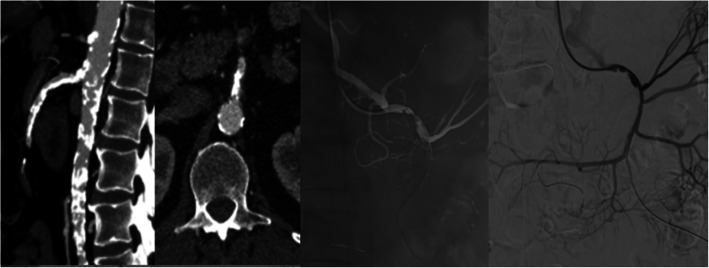
CTA revealed highly calcified stenosis in the proximal segment of SMA. Selective brachial access angiography confirmed severe SMA stenosis

The symptoms of the 67-year-old man included 9 months of postprandial abdominal pain, most severe in the last 2 months and 10 kg of weight loss. His comorbidities include diabetes and revascularized ischemic cardiomyopathy 7 years ago. CTA revealed an extensive atherosclerotic disease involving aortoiliac, celiac and SMA calcification. Selective angiography revealed severe stenosis in the celiac and SMA, with hepatic artery originating in the same SMA **(**Fig. 1**).** The choice of treatment was a multidisciplinary team decision due to the calcification. We confirm the written informed consent of the patient before the start of treatment.

**Fig. 2:**
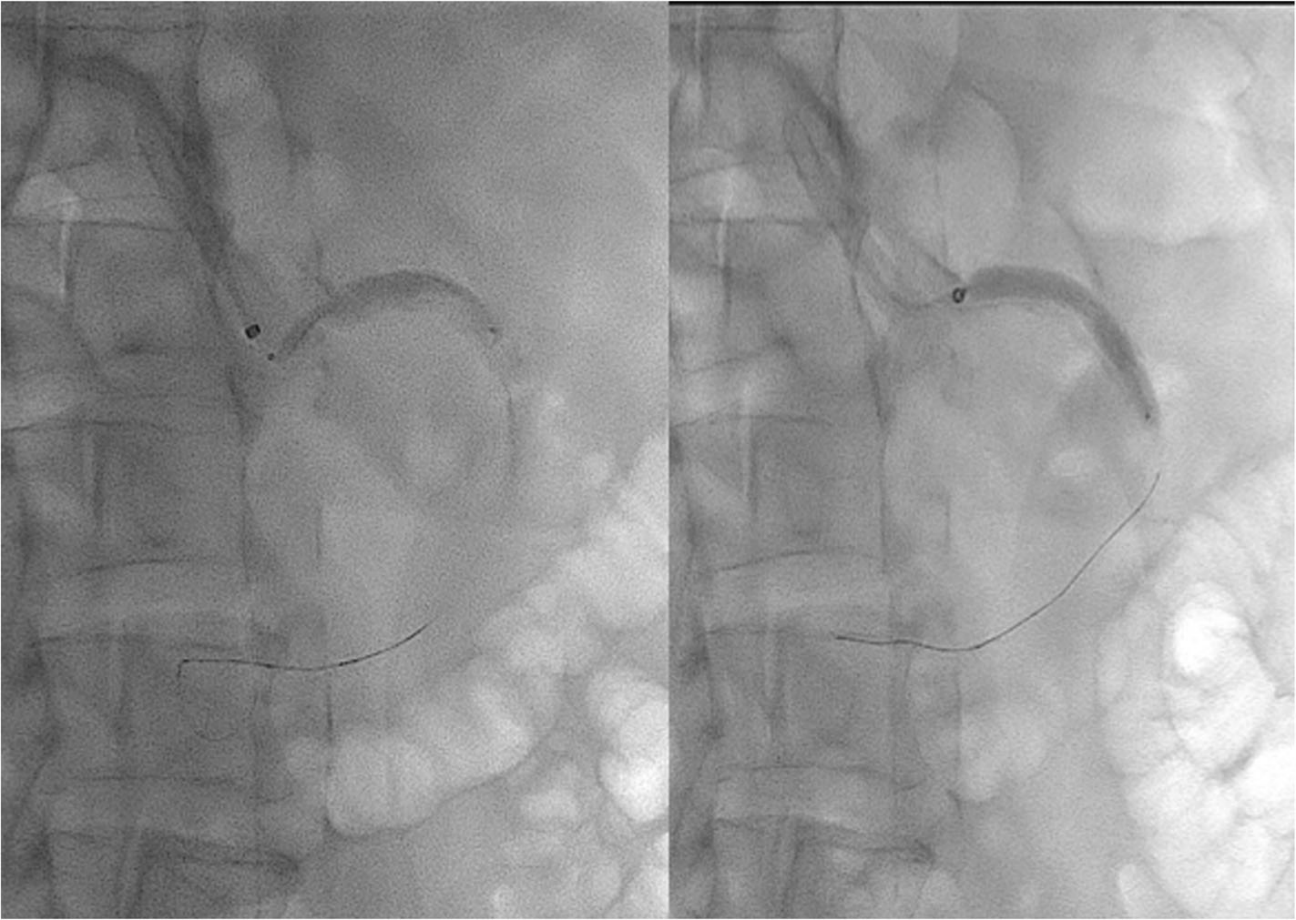
Shockwave Lithotripsy M5–6 × 60 mm, balloon catheter placed first through the SMA ostium, and total subsequent coverage of stenosis. Balloon inflated to 4 atm during energy emission

**Fig. 3:**
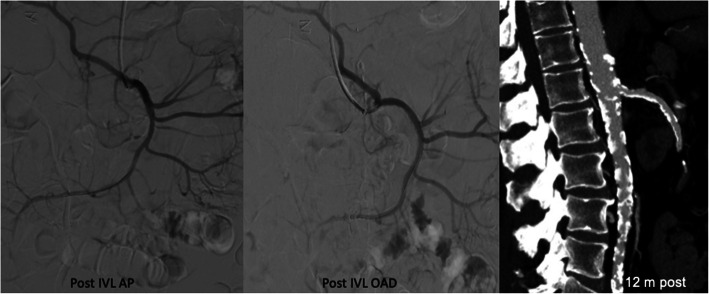
Control arteriography post-treatment with IVL, and MPR of CTA 12 months post-treatment

The patient was on antiplatelet therapy. Percutaneous endovascular intervention of SMA was performed under sedation. Vascular access was established in the right brachial artery with 6 Fr flexor guide sheath 80 cm long (Cook Medical, Bloomington, IN, USA) advanced towards the abdominal aorta with distal end in front of the SMA origin. A 110 cm long Shockwave Lithotripsy M5 6 × 60 mm balloon catheter (Shockwave Medical Inc., Santa Clara, California) over Traxcess 14 guidewire (Microvention-Terumo Inc., California, USA) was then placed through the SMA ostium, and 5 of 1-pulse treatments were delivered to activate only the distal emitter and facilitate calcium fragmentation and advancement of the IVL balloon catheter **(**Fig. 2**).** Once the balloon catheter had been advanced and covered all stenosis, 5 complete cycles of 30 pulses were applied each cycle with good angiographic result (Fig. 3**).** The patient tolerated the procedure without complications and went home 24 h later. A month later he reported no complications after percutaneous endovascular intervention and absence of abdominal pain. Twelve months after the procedure, the patient gained weight and has not complained again of postprandial abdominal pain (Fig. 3**)**.

## Discussion

Treatment of symptomatic CMI is widely accepted to prevent acute mesenteric ischemia. There is no documentation on the use of IVL in the treatment of atherosclerotic disease presenting as mesenteric ischemia. Open surgical repair has survival rates of approximately 80% to 90% (Lejay et al. 2015). Intravascular treatment has become an alternative to open surgery. One study showed that although primary patency rates were below 45%, secondary patency rates were comparable to 94%. The presence of highly calcified ostial occlusions represents an important limitation of endovascular interventions (Van Petersen et al. 2010).

The Shockwave Lithoplasty® System IVL consists of three components: the balloon catheter for performing the IVL, the connecting cable for pulse therapy, and a power generator. The IVL catheter consists of an OTW balloon catheter, which has, inside, several emitters of sonic shock waves whose diffusion generates a pressure of 50 atm. Activation of the device applies pulsed mechanical energy transmitted in the form of shock waves that pass through soft tissue without altering it and without causing damage to the vessel. Upon reaching the calcified plaque, the transmitted energy generates microfractures, modifying the calcium and therefore the morphology of the artery, allowing a posterior dilation with the balloon at low pressure (6 atm).

The application of IVL technology has been reported in coronary and peripheral endovascular procedures, including the iliac, femoral, and renal arteries. Currently, several multicenter studies are being conducted to evaluate the safety (DISRUPT CAD II) and efficacy (DISRUPT CAD / PAD III) of IVL in the coronary and peripheral territories (Adams et al. 2020; Brinton et al. 2019; Brodmann et al. 2017; Armstrong et al. 2020). The first studies on its peripheral use show promising results confirming a patency of 100% after 30 days, 70–80% at 6 months and 54–70% at 12 months, with a 95% clinical success rate, indicating less than 50% residual stenosis after the procedure with no evidence of acute adverse cardiac events, embolizations, thrombus formations, or perforations (Brinton et al. 2019; Brodman et al. 2019).

This report with the application of IVL therapy demonstrates successful revascularization in lesions that combine calcium thickness and a large calcium arc (270° of the vessel circumference), making dilation difficult with conventional therapy.

## Conclusion

IVL is an emerging treatment modality that dilates calcified lesions endovascularly. Its use in the treatment of highly calcified SMA in patients with postprandial pain should be considered as a novel approach for patients whose traditional endovascular treatment modalities are not optimal. More controlled studies are needed to demonstrate the efficacy, safety, and feasibility of this new technology.

## Data Availability

Not applicable.

## Abbreviations

CMI: Chronic mesenteric ischemia
SMA: Superior mesenteric artery
CTA: Computed tomography angiography
PTA: Percutaneous transluminal angioplasty
IVL: Intravascular lithotripsy
